# Reflections on a journey: a retrospective of the ISCB Student Council symposium series

**DOI:** 10.1186/s12859-018-2369-x

**Published:** 2018-10-09

**Authors:** Mehedi Hassan, Aishwarya Alex Namasivayam, Dan DeBlasio, Nazeefa Fatima, Benjamin Siranosian, R. Gonzalo Parra, Bart Cuypers, Sayane Shome, Alexander Miguel Monzon, Julien Fumey, Farzana Rahman

**Affiliations:** 1 Genomics and Computational Biology Research Group, Faculty of Computing, Engineering and Science, University of South Wales, Cardiff, UK; 2 Luxembourg Centre for Systems Biomedicine, Université du Luxembourg, Belvaux, Luxembourg; 3 Computational Biology Department, Carnegie Mellon University, Pittsburgh, Pennsylvania USA; 4 Department of Biology, Faculty of Science, Lund University, Lund, Sweden; 5 Department of Genetics, Stanford University, Stanford, California USA; 6 European Molecular Biology Laboratory, Heidelberg, Germany; 7 Institute of Tropical Medicine and the University of Antwerp, Antwerp, Belgium; 8 Bioinformatics and Computational Biology Program, Iowa State University, Ames, Iowa USA; 9 Structural Bioinformatics Group, Departamento de Ciencia y Tecnología, Universidad Nacional de Quilmes, Buenos Aires, Argentina; 10 Human Genetics and Cognitive Function, Institut Pasteur, Paris, France

**Keywords:** ISCB, Student Council, Symposium, Networking, Leadership, Young, Computational Biology, Career, Development, Science Communication, Community, ISMB, ECCB, SCS

## Abstract

This article describes the motivation, origin and evolution of the student symposia series organised by the ISCB Student Council. The meeting series started thirteen years ago in Madrid and has spread to four continents. The article concludes with the highlights of the most recent edition of annual Student Council Symposium held in conjunction with the 25th Conference on Intelligent Systems for Molecular Biology and the 16th European Conference on Computational Biology, in Prague, in July 2017.

## Introduction

The Student Council of the International Society for Computational Biology (ISCB) is a student body, composed of volunteer students and post-doctoral researchers. The council started its journey in 2003, with the primary goal of fostering the development of the next generation of computational biologists. The board of directors of the ISCB officially approved the Student Council in July 2004 at its annual meeting on *Intelligent Systems for Molecular Biology* (ISMB) in Glasgow, Scotland [1].

The ISCB Student Council (ISCB-SC) endeavours to meet its goals through a wide range of activities, namely, holding annual symposia [2], arranging internships for students from around the world to work in well-established labs [3] and forming a global network of Regional Student Groups (RSGs) [4]. Together with colleagues in 33 regions across the globe, members of the ISCB-SC devote their time in hosting events such as webinars [5, 6], workshops, and lectures-therefore, providing networking opportunities and promoting the development of a global computational biology community. Another significant role the ISCB-SC plays is to represent the needs of students and post-doctoral researchers to the broader community, through the ISCB.

The range and diversity of the ISCB-SC activities have grown significantly in recent years; nevertheless, the annual symposium remains to be the flagship event and the guiding activity, where colleagues from all corners of the globe congregate to share their research and ideas.

## The origin of ISCB Student Council Symposium

The Student Council Symposium (SCS), modelled after the large-scale international conferences that are common in academia, provides a crucial step in organisational and leadership development of young researchers [2]. The meeting also serves as a platform for participants to hone their skills in presenting their research before sharing it at large conferences.

The founders of the ISCB-SC recognised the importance of attending larger meetings, such as the annual conference on *Intelligent Systems for Molecular Biology* (ISMB) and the *European Conference on Computational Biology* (ECCB). A large academic conference is not only place to practice science communication skills but also a platform to disseminate exciting research to the peers. However, through their own experience they realised that for a student or an early career researcher, it could be a daunting and overwhelming task to present at large conferences in front of an audience of field experts and potential future employers. To help students address these challenges, the early leaders of the student council initiated a symposia series ‘for the students, by the students’. The very first SCS took place in Madrid, Spain on September 28, 2005, [2, 7] directly preceding the 4th ECCB. The inaugural symposium consisted of 19 student presentations and one keynote presentation.

The success of the first SCS led to the introduction of a yearly event held in the days before ISMB.

### Versions of the SCS

Over the past two decades computational biology has transformed from being a misunderstood field [8] to being enthroned as the pillar of new biology [9]. The availability of ‘omics data at a growth rate faster-than-Moore’s-Law [10] inspired the launching of increased numbers of scientific projects focusing computational biology, leading to an apparent explosion of career opportunities [11]. The ISCB-SC recognised a growth in the number of RSGs across the globe and saw the need to host more than one SCS, preferebly in different geographic regions. This was also influenced by the schedule of ISCB meetings in different parts of the world.

ISMB alternates its venue between Europe and North America each year, and in every two years both conferences take place together in Europe as ‘ISMB/ECCB’. There are also biennial meetings by the ISCB in Latin America and Africa.

After five successful SCS the ISCB-SC planned to bring regular events closer to the student hubs and in 2010, the society introduced the ‘first European Student Council Symposium (ESCS)’ [12] which was the first SCS to precede ECCB since SCS in 2005. In 2013, plans for further editions in Latin America (SCS-LA) and Africa (SCS-Africa) were initiated.

**Fig. 1:**
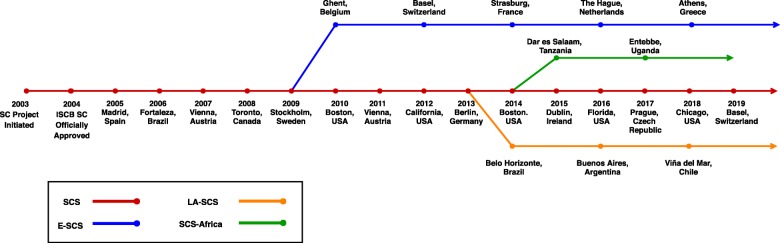
Timeline of symposia series by the ISCB Student Council Different editions of Student Council Symposium with their locations per year. Dots on the central red line denotes years. Three branches represent three regional editions of SCS, they are: Blue for ESCS, Orange for LA-SCS and Green for SCS-Africa

In 2014, for the first time three versions of SCS took place in the same year: SCS (USA) [13], ESCS (France) [14] and the first LA-SCS (Brazil) [15]. The first edition of the SCS-Africa took place in Tanzania in 2015 [16] followed by in Uganda in 2017 [17]. Currently, all three regional versions of SCS are biennial events (see Fig. 1 for a detailed timeline).

**Fig. 2:**
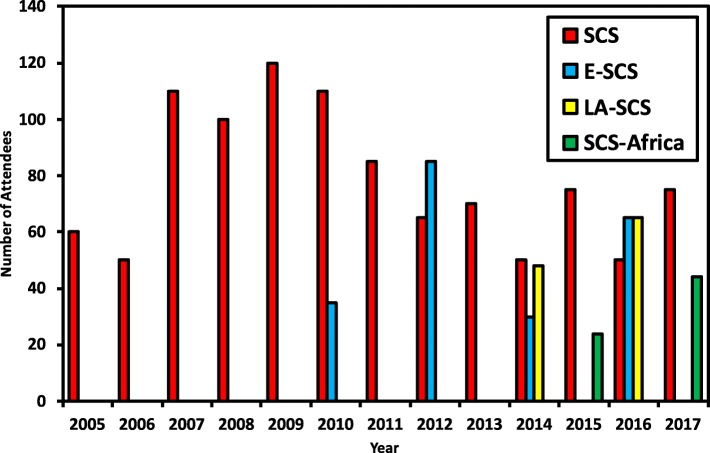
Statistics of attendees per symposium The chart shows the distribution of attendees across various editions of the SCS. Colour legends are: Red bar for SCS, blue for ESCS, yellow for LA-SCS, and green for SCS-Africa

Although all the regional versions of SCS provide the same format of scientific and social settings, higher attendance has been observed at the SCS when the event takes place in Europe (ISMB/ECCB) than the US-based editions of the meeting [18]. Figure 2 shows the yearly attendees number at SCS, ESCS, LA-SCS and SCS-Africa.

### What role does a symposium play?

The goal of SCS events is two-fold: to prove participants with an opportunity to participate in an academic meeting with a lower pressured environment; and to provide organisers with a chance to gain a first-hand leadership experience which they may not otherwise get until in the later stages of their careers.

Oral presenters at SCS benefit from being able to describe work that are not yet ready for larger conferences. By centring a meeting around students and post-doctoral researchers, this work may get more feedback than it may have from a poster alone at a major academic conference. For those who have never presented their work before, it gives them an opportunity to present in a less stressful environment while still being a highly beneficial experience. Attendees who are not selected for oral presentations also benefit from seeing work presented in early stages; additionally, they are more likely to ask questions without fear of being embarrassed in front of more experienced researchers.

All participants are encouraged to present posters at SCS. Some delegates present the same work on the main ISCB conference following a presentation at the SCS. For these attendees, the SCS poster session is a chance to practice in a more relaxed atmosphere. Just as with oral presenter, those who have not presented a poster at a conference before will ideally be more comfortable in future poster sessions.

## Organising a symposium

In 2008, organisers of the student council published an editorial to help young and relatively less experienced organisers successfully put together a student symposium. Till date, the organising of ISCB Student Council Symposia follows the same general trends outlined by [19]; but as the SCS has matured, so too have the associated policies and procedures. The planning and brainstorming for the next symposium begin immediately after the yearly event. The ISCB-SC’s Executive Team members seek an organising team (chair, co-chair) for the upcoming events. To help the council find as many new leaders as possible among young researchers, new faces are encouraged to participate in symposium organising committees.

Driven by the expected outcome and inspired by the previous experience, a set of committees are organised to steer activities that are required to accomplish the tasks: abstract collection and review, publicity, financial planning and spending, as well as many others. New volunteers are encouraged to participate in all sub-committees and also pitch new ideas to make the event more successful.

Commonly formed committees are:

- Programme Committee,
- Finance Committee,
- Outreach (Communication) Committee,
- Travel Fellowship Committee,
- Web Committee.

### The format of the symposium

Since inception SCS and its derivatives have evolved to include a diverse set of activities. Still revolving around the core structure of an academic meeting (namely research presentations, keynote speakers, poster sessions, and networking) the format now often includes activities that are unique to SCS such as an ice-breaking session, career panel sessions, and short-form lightening-fast or flash talks.

The day usually starts off with a quick ice-breaking session where delegates have an opportunity to introduce themselves and their research interests to each other in a completely informal setting.

A select group of students are invited to present their work as a typical long-form talk (10-15 min) followed by a question and answer section (usually 3-5 min). From SCS 2014, some SCS events also contained a flash talk format where students get an opportunity to present their work in a more compressed time slot (less then 3 min). The flash talks are a derivative of ‘elevator-pitch’ or techno-pitch’ [20]. These talks are meant to be an informal way to gather interest for more detailed conversations during the poster sessions (often referred to as ‘oral posters’). The success of flash talks rests in summarising exciting findings of research quickly and succinctly, a skill which is difficult to learn without practice.

The day often includes a session where industry partners present on topics which may be of interest to the students in attendance.

While keynote talks from senior community members are the hallmark of almost all academic meetings, the keynotes at SCS are encouraged to not only share stories of their academic achievements but also provide advice and insights into the life in academia. This means that while the keynote speaker may overlap with the keynotes at the main meeting, the talk at the SCS often has a different focus.

### Abstracts and peer review

In this shorter format academic meeting, it is crucial to maintain a high bar on the quality of the works presented. Academic peer-review best practices guide this aspect. Each year, an abstract call is opened few months before the symposium and abstracts are received from the student authors. A select group of volunteer members then review the abstracts, typically those who are at a later stage of research degrees or in their early post-doctoral training years.

Like most aspects of the symposium, the focus is on the development of our peers. Therefore, the reviewers are asked to screen for abstracts which are suitable for the symposium and make recommendations whether to invite the authors for an oral presentation. The SCS usually calls for a minimum of two reviews and where applicable this is increased to three or more reviews for each submitted abstract. Similar to a process used for academic journals, reviewers declare their areas of interest and the program committee assigns abstracts in those areas. To avoid unintended bias [21, 22] the SCS events use a custom built abstract review system that utilises double-blind peer review [23] of abstracts.

### Travel fellowships

In many cases, students and early-career researchers struggle to attend conferences and symposia on a shoestring budget. Offering them fellowships to assist in their journey is much appreciated by participants, and, in some cases essential. At the SCS events, these are awarded through competitive reviews of submitted abstracts and a candidate’s motivation for participation. The fellowships empower students to present at the symposium and also motivates to contribute back to the community. Some current and past leaders of the council were travel fellowship awardees.

Financial support such as travel grants are particularly beneficial for students studying in developing countries. These opportunities allow students to build upon their academic experiences and give them a chance to participate in a networking environment through discussions with colleagues from the global community.

**Fig. 3:**
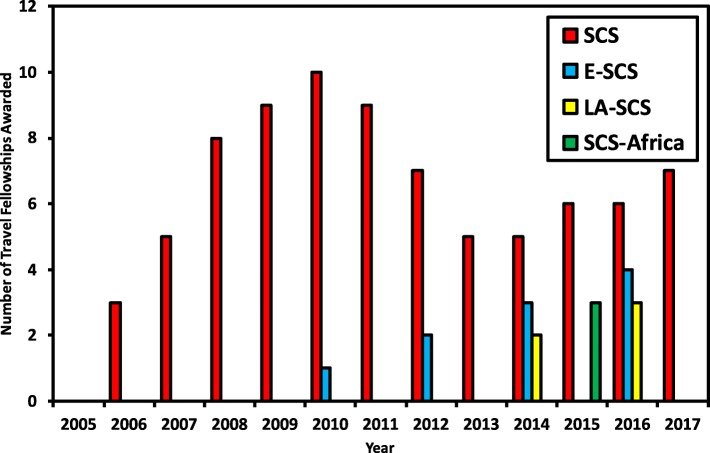
Statistics of travel fellowships awarded per symposium The chart shows the number of travel fellowships in SCS and its derivative events. Colour legends are: Red bar for SCS, blue for ESCS, yellow for LA-SCS, and green for SCS-Africa

The second SCS was the first to offer dedicated travel fellowships in 2006. Between 2006-2017, a total of 80 travel fellowships were awarded in SCS events and 18 fellowships were given in regional SCS events (see Fig. 3 for details).

The amount and number of awards vary based on funding raised from sponsors in any given year. A comparison of charts from Figs. 2 and 3 shows a trend where in years when a lower number of travel fellowships were awarded, the number of attendees was smaller too. The number of awarded fellowships directly corresponds to the amount of raised funds, and the fund-raising often relates to the number of active volunteers available; in retrospect it can be assumed that in the years with lesser attendees, there were fewer volunteers working to promote the event (lower attendees) and securing lower funds (fewer fellowship winners). It is possible that it was a coincidence or weak correlation; however, this is supported by observations of recent years.

The financial assistance requested by attendees is often higher than what the Student Council can cover. This is reflected in the fact that the number of applications received is much higher than the number of available fellowships. For example, a total of 40 applicants applied for travel fellowships to attend the SCS 2017, but there were only seven fellowships available to be awarded.

Due to a large number of submissions, the fellowship applications need to be reviewed very carefully. To ensure the available funds have the most impact, the SCS travel fellowships reviewers consider the career level, motivation, abstract quality, current academic activity/exposure and community commitments. The review process follows a scoring matrix to evaluate the application against the aspects mentioned above. A fellowship is only offered to presenting authors attending the symposium.

The review panel usually consists of early post-doctoral researchers, Student Council executive team members, and symposium organisers who are not eligible to apply for a fellowship. The Student Council is continuously considering further improvements and streamlining for the travel fellowships review process.

Many times unique situations arise that might not conform to the scoring rubric we have defined and, therefore, require special considerations to the process. In 2015, for the 11th Student Council Symposium, the ISCB Student Council received an outstanding application from a high school student with great motivation requesting a travel fellowship. In this case, the applicant received an especially considered ‘Inspiring Youth Travel Fellowship’ [24].

The success of SCS travel fellowship scheme has been possible due to our generous contributors. As a student-led organisation, the ISCB Student Council relies on support from its parent organisation the ISCB, higher education institutions, bioinformatics companies, and organisations to fund these awards.

### Financial planning

One of the significant factors of a successful symposium is fundraising. The ISCB and participant registration costs support some core costs of organising the event in the form of venue and refreshments. The Student Council relies on sponsorships to cover remaining costs.

A critical cost item for organising the event are the travel fellowships awarded to students who are in need of financial assistance to attend the meeting. Beyond the travel fellowships, there are other costs including the awards for best talk and poster, costs associated with the printed material, symposium report publications and off-venue networking sessions.

The fundraising practice is a vital aspect of the transferable skills development of students as the organisers learn various elements of commercial activities, such as creating a budget, designing promotional materials, budgeting, contract negotiation, and spending management.

At the beginning of the organising year, a finance committee prepares a draft budget and sponsorship package. Volunteers then approach organisations intending to source sponsorship and negotiate terms. Research students, who otherwise have limited exposure to commercial communications and negotiations, benefit from this exercise.

Maintaining a good rapport with the network of past sponsors, and working with them on ideas to improve upcoming events, helped us build consistent collaborations with many companies and institutes. Although acquiring funds is essential to run the symposium, it comes with the challenge of balancing the commercial and scientific priorities. While we strive to meet expectations of our sponsors, we always maintain the prime target of keeping the symposium an academic event for the benefit of students. Hence, past editions saw commercial partners delivering talks on cutting-edge technologies, educational courses, and career opportunities.

It is, therefore, paramount in sponsorship or contracts negotiation that from the beginning the student volunteers establish expectations as well as the symposia scope. These activities help student volunteers gain essential leadership skills.

It is worth noting that a few long-standing supporters of the SCS helped the event gain momentum over the years. This list includes, but is not limited to, the Oxford University Press, Elsevier, The Swiss Institute of Bioinformatics (SIB), Harvard Medical School, and the Earlham Institute (previously known as The Genome Analysis Centre).

Oxford University Press has been a long-standing sponsor of Best Presentation and Poster Awards at SCS. The Swiss Institute of Bioinformatics has, on several occasions, supported the SCS by sponsoring Travel Fellowships.

### Publications

Since the third SCS [1], the highlights of meetings were published in peer-reviewed journals in the form of editorial or a meeting report. In recent years, some highlights were published in non-peer reviewed journals [25]. These highlight articles form a record of the successes of each additional year and shine a spotlight on the newest researchers in the field.

## Highlights of the Student Council Symposium 2017

The 13th edition of SCS took place in Prague, the Czech Republic on July 21, 2017, directly preceding the joint conferences of 25th ISMB and 16th ECCB [26]. This year, 70 delegates attended from all over the world. The day consisted of 9 full-length talks and 14 flash talks, 60 poster presentations, and 2 keynote speakers.

Talks were divided into three themed sessions: (i) Proteins, DNA, Sequences and Evolution; (ii) Networks and 3D Interactions; and (iii) Modeling Cancer, Viruses and Diseases. An industrial speaker presented a workshop on biological pathway analysis tool Pathway Studio ^*Ⓡ*^ [27, 28].

Another industrial speaker, Dr Fiona Nielsen from Repositive.io, delivered an inspirational career-centric talk titled “A bioinformaticians spark which leads to a new platform for sharing genomic data”.

### Keynotes

Professor Christine Orengo, from University College London, delivered the first keynote address at the SCS 2017. Prof. Orengo is renowned for her contribution to protein structure prediction work, particularly the CATH database [29]. The main topics that the Orengo team focuses on is the study of protein evolution and the design of algorithms to aid in protein studies. Prof. Orengo discussed an algorithm that identifies diverse superfamilies to trace back the last universal common ancestor (LUCA), even when relatives have diverged considerably to acquire modified structures and functions. She also highlighted that phylogenetic analyses of protein families can provide insight into the evolution of novel substrate specificities, and that functional studies can be combined with thermodynamic analyses to reveal the energetic considerations associated with functional divergence.

The second keynote was given by Dr. Johannes Söding who is known for his work on protein homology detection and protein structure prediction. At SCS 2017, Dr. Söding presented two newly developed tools in his lab which aim to solve some of the most prominent challenges in metagenomics [30]. MMSeqs2 performs profile-based searches for homologous proteins in large metagenomic databases with more sensitivity than PSI-BLAST and at more than 400 times its speed. To build such profiles, clustering is needed first. LinClust, part of the MMSeqs suite, helps to cluster billions of protein sequences in linear time. As a working example, 1.7 billion sequences from 2200 metagenomic and metatranscriptomic datasets were clustered by LinClust in 30 hours on 28 cores.

### Student presentations

Young researchers travelling from as far as Argentina and India presented topics ranging from protein sequence evolution to predicting copy number variants in cancer. Judging by student votes, Rodrigo Gonzalo Parra’s talk titled, “*scTree: reconstructing complex cellular lineage trees from single-cell RNA-seq data* [31]” was the favourite. His work describes a method to understand the emergence of cellular lineages from complex single-cell RNA-seq experiments. A recording of the talk is available online on the Student Council’s YouTube channel [32].

In the flash talks session, two talks stood out from the rest. Joske Ubels, a PhD student from University Medical Center Utrecht, explained her research with creative cartoons and easy to understand graphics. The exact method behind “*TOPSPIN: a novel algorithm to predict treatment specifical survival in cancer* [33]” was explained in detail at her poster later in the day.

Taking a slightly different route, Gal Hyams from Tel Aviv University presented a series of “buzzwords” related to his research. He closed with, “To understand the common link, you have to visit my poster: *CRISPys: gene family silencing using the CRISPR-Cas9 system* [34]”. Judging by audience applause, this novel approach was well received! All of the flash presentations are available online on the Student Council’s YouTube channel [35].

## Next editions

Following on from the success of the 13th SCS in 2017, the ISCB-SC successfully hosted the 14th ISCB Student Council Symposium in Chicago, USA [36] on July 6, 2018. The regional editions of the symposia series will begin with ESCS in Athens, Greece on September 8, 2018 [37]; followed by the LA-SCS in Chile, on November 9, 2018 [38]. Plans for the next edition of the SCS have already began and the event will take place in Basel, Switzerland in 2019 [39].

## Outlook

More than a decade since the inaugural symposium, the ISCB Student Council Symposia continue to be successful and impactful platform attracting participation from aspiring researchers from all over the world. Attendees responded well to the inclusion of flash talks, the addition of recorded talks, and the continuation of a long line of high impact keynotes.
